# A systematic review of family carers' experience of compassion when caring for older adults

**DOI:** 10.1186/s12877-026-07523-6

**Published:** 2026-04-27

**Authors:** Joel F. Barnett, Nicola White, Laura Dillon, Pepsi Reilly, Elizabeth Sampson, Claudia Cooper, Nuriye Kupeli

**Affiliations:** 1 Marie Curie Palliative Care Research Department, Division of Psychiatry, University College London, London, UK; 2 Centre for Psychiatry and Mental Health, Wolfson Institute of Population Health, Queen Mary University of London, London, UK; 3 Academic Centre for Healthy Ageing, Whipps Cross Hospital, Barts Health NHS Trust, London, UK

**Keywords:** Carers, Compassion, Review, Adults, Dementia

## Abstract

**Background:**

Although caring can be a rewarding experience, family carers of older adults frequently report isolation, stress, anxiety, and burden, which may hinder their ability to cultivate compassion for self and others. Cultivating compassion is associated with better psychological health and wellbeing and may therefore act as a protective factor against the adverse emotional impacts of caregiving.

**Objectives:**

This review aims to: (1) understand carers’ experiences, including the barriers and facilitators to cultivating and maintaining compassion when caring for older adults; (2) identify the tools that have been used to assess the experience of compassion in carers and to determine if they have been developed with this population; (3) explore if compassion as experienced by carers is related to other health and wellbeing outcomes.

**Methods:**

We conducted a search across five electronic databases from inception to December 2025. Eligible peer-reviewed studies reported qualitative and/or quantitative data on experiences of compassion among family carers of older adults (aged > 65 years), or people living with dementia. Study quality was appraised using the Hawker tool. We used a narrative approach to synthesise the qualitative and quantitative data.

**Results:**

Twenty-nine papers reporting 28 studies were included. Qualitative synthesis identified compassion as primarily other-directed, relational, and grounded in love, moral obligation, and identity. Compassion was facilitated by attachment, meaning, and alignment with carers’ values, but undermined by emotional over-identification, chronic responsibility, fatigue, guilt, and limited support. Quantitative studies used a wide range of compassion-related constructs and measures, none of which were developed with carers. Quantitative evidence showed consistent associations between higher self-compassion and better psychological wellbeing, adaptive coping, and lower burden. Findings for compassion directed toward others were mixed, with emotionally over-engaged forms associated with distress, while motivational or value-based compassion was linked to more positive caregiving appraisals.

**Conclusions:**

Compassion in caregiving is a complex and relational phenomenon that can be both sustaining and burdensome. While self-compassion and receiving compassion from others appear protective, unbuffered compassion for others may increase vulnerability to distress. Future research should prioritise longitudinal designs, carer-specific measures of compassion, and interventions that support sustainable, relationally grounded compassion.

**Trial registration:**

PROSPERO CRD42019134233, registered 22 May 2019.

## Background

A combination of an ageing baby boom generation and advances in modern medicine has resulted in an increasingly ageing society and a rise in the number of people living with progressive and life-limiting conditions such as dementia. Dementia is a progressive, neurodegenerative disease and the leading cause of death in the United Kingdom (UK) in 2025 [1] with recent estimates suggesting that by 2040, there will be 1.4 million people living with dementia in the UK [2]. Keeping people living with dementia (PLWD) at home for longer and shifting care from hospitals to the community are key governmental goals [3, 4] and while this has benefits for the PLWD, and wider society, such as improved quality of life and lower care costs, respectively [5], this care predominantly falls on family or friend carers (referred to as carers hereon).

### The impact of caring

In the UK, there are 5.8 million carers [6] of whom over 790,000 people care for someone living with dementia with this number estimated to rise to over a million by 2040 [7]. Caregiving responsibilities of older adults commonly mean family, friends and communities come together to support [8] and enable older adults to live independently for as long as possible. While caring can be a positive experience, including an enhanced relationship with the care recipient and a sense of accomplishment, personal satisfaction and personal growth [9], carers report that providing care takes over their life and that they are unable to detach from their caring responsibilities [10]. Carers can become increasingly isolated from family and friends [11] and, unlike health care professionals, may not be able to take time off [12, 13]. Carers report experiencing stress, depression, anxiety, grief and burden [14–17] and around half have been reported to exhibit some potentially harmful behaviour towards the person they are caring for [18]. Research has shown that the effects of caring on carer health changes across the disease trajectory based on the level of care the care recipient requires ranging from anxiety and burden during the earlier phases of the disease to distress and complicated grief before, during, and after bereavement [19]. One potential approach is to develop interventions that support carers in cultivating compassion to help regulate emotions during times of difficulty.

### Compassion

The literature includes a number of terms that refer to compassion and thus various definitions have been proposed [20]. This review is interested in all terms that describe the experience of compassion which includes, but is not restricted to self-compassion, compassion for others, receiving compassion, compassion satisfaction, compassion fatigue (sometimes referred to as empathic distress [21]) and compassionate love. While these terms should not be used synonymously or interchangeably, they provide a framework for understanding of how compassion is experienced.

Compassion for self and others has been defined as the “sensitivity to suffering in self and others, with commitment to alleviate and prevent it” [22] and “a distinct affective experience whose primary function is to facilitate cooperation and protection of the weak and those who suffer” [23]. While compassion fatigue has been likened to vicarious trauma, burnout and burden [24] and defined as “a state of exhaustion and dysfunction biologically, psychologically, and socially as a result of prolonged exposure to compassion stress” [25]. These definitions encompass both cognitive and behavioural processes, including the recognition of suffering, an understanding that suffering is a universal human experience, the capacity to emotionally resonate with suffering while tolerating difficult feelings, and a motivation to alleviate suffering [20]. It is important to note that compassion is distinct from related constructs such as empathy [26], with evidence indicating the activation of specific neural systems when compassion is experienced [27]. In essence, empathy involves sharing or understanding another’s emotional state, whereas compassion incorporates a prosocial motivation to alleviate suffering, supported by neurobiological patterns linked to affiliative and caregiving systems [26].

### Caring and compassion

A number of reviews have synthesised literature on compassion as a broad concept in carers and older adults [12, 28, 29] and with a focus on self-compassion [30, 31] and compassion fatigue and satisfaction (based on our understanding of compassion fatigue in healthcare professionals [32–35]). In this review, we update and extend past syntheses. We aimed to examine measured in carers of older people, including people living with dementia, and if any of the tools used were developed in this population; to report any associations with health and wellbeing outcomes; and to synthesise qualitative studies in this population to explore carers’ perceptions of compassion, how it is enacted in caregiving experience, and what enables or constrains its maintenance over time.

### Aims

The aims of this review are to:

1. Understand carers’ experiences of compassion when caring for older adults, including understanding of compassion and the barriers and facilitators to cultivating and maintaining compassion
2. Identify the tools that have been used to assess the experience of compassion in carers and to determine if they have been developed with this population
3. Explore if compassion as experienced by carers is related to other health and wellbeing outcomes

## Methods

This systematic narrative review follows the Preferred Reporting Items for Systematic Reviews and Meta-Analyses (PRISMA [36]) and a protocol for this work is registered on PROSPERO (CRD42019134233). During the early stages of data extraction and preliminary screening, it became clear that carers’ experiences of compassion varied substantially depending on the condition, age, and needs of the care recipient. To address this heterogeneity and maintain conceptual coherence, we refined the focus of the review to carers of older adults. This adjustment enabled a more clearly defined population and strengthened the interpretability of the findings. Although this represents a deviation from the broader caregiver population outlined in the registered protocol, it did not alter the overarching aims, research questions, or methodological processes of the review.

### Search strategy

#### Searches were conducted in five electronic bibliographic databases including the Cumulative

Index to Nursing and Allied Health Literature (CINAHL), EMBASE, PsycINFO, Medline and the Cochrane Central Register of Controlled Trials (CENTRAL). Citations indexed from inception of each database to 5th December 2025 were identified. A combination of Medical Subject Headings (MeSH) and free text terms on compassion and carers of older adults living with dementia were used. Terms for compassion and carers were developed from terms used in previous reviews [8, 20, 37]. See Table 1 for an example of the search strategy used. Backward and forward citation searching was undertaken to identify additional studies.

**Table 1:** Example search strategy

#1 (care-giver* or caregiver* or care giver* or carer* or daughter* or son*or dependent* or families or family* or folk* or kinship or parent* or relative* or spouse* or wife* or wive* or husband* or Carers)
#2 (Compassion* or “self-compassion” or “compassion-focussed” or “compassion-focused” or “compassion fatigue” or empath*or kindness or sympath* or mindful* or Empathy)
#3 (Aged or “old people” or “older people” or “old person” or older persons or old resident* or older resident* or elder* or geriatric or “wernicke encephalopathy” or “cognitive disorders” or dement* or alzheimer* or lewy bod* or chronic cerebrovascular or “organic brain disease” or pick* disease or huntington* or binswanger* or korsako* or “Dementia” or “Aged”)
#4 1 and 2 and 3

### Eligibility criteria

Studies were included if they: (1) explored experiences of compassion (including compassion for the self and others, receiving compassion, compassion fatigue and compassion satisfaction) from the perception of an informal or unpaid carer of an older adult (65 + or if the mean age of the sample was 65 +) or at least 50% of the sample is caring for someone living with dementia; (2) were peer-reviewed primary research studies reporting qualitative and/or quantitative data (including RCTs, experimental studies, interview studies where compassion scores are reported); (3) were published in English. We excluded studies where the age of the care recipient was not reported or specified as an inclusion/exclusion criteria for recruitment (and the population did not have dementia). We excluded protocols, dissertation theses and conference abstracts.

### Study selection

The process for selecting suitable studies was conducted by four independent reviewers for all citations (NK, NW, PR and LD). The first stage involved assessing titles and abstracts using the eligibility criteria followed by a full-text review of all studies that met the inclusion criteria or where relevance was unclear. If access to the full-text of a study was not possible or if following full-text review eligibility of a study was still unclear, then the authors were contacted for more information. Disagreements on relevance of a study were discussed and resolved through consensus.

### Methodological quality assessment

The Hawker [38] quality appraisal tool was used to assess the methodological quality of the included studies. This checklist is designed to assess and score quality of both qualitative, quantitative and mixed methods studies. The nine sections of checklist cover: title and abstract, introduction and aims, method and data, sampling, data analysis, ethics and bias, findings and results, transferability and generalisability and implications and usefulness. Each section is rated as good, fair, poor or very poor and an overall score is calculated. Overall scores represent poor (⩽18), fair (19 to 27) and good (⩾28) [39, 40] methodological quality. Each included study was quality appraised by two independent reviewers (NK, NW) and any discrepancies in ratings were discussed and resolved through consensus. Due to the exploratory aim of the review, no studies were excluded based on methodological quality.

### Data extraction

Data from each included study were extracted by two independent reviewers (NK and NW) using a standardised form, including: study characteristics (author, year, country, aims), sample characteristics (age, gender, ethnicity, education, marital status, relationship to older ageing adult, time caring) and methodology (study design, sample size, recruitment process, setting). Data on compassion included the concept explored (i.e., compassion for self, compassion for others, compassion from others, compassion satisfaction, compassion fatigue) and where quantitative data were reported, means, standard deviations, correlations and other statistical data were extracted. Where data from studies reporting on interventions and Randomised Controlled Trials (RCTs) are included, only baseline data was extracted. Where studies reported qualitative data, the results section of each paper was extracted including participant quotes and corresponding author interpretations.

### Data analysis and synthesis

A parallel narrative approach as described by Popay et al. [41] was adopted for analysing the qualitative and quantitative data. We sought to understand carers’ experiences of compassion when caring for older adults, including understanding of compassion and the barriers and facilitators to cultivating and maintaining compassion and determine the relationship between compassion and other health and wellbeing outcomes, respectively. For the qualitative synthesis, we followed a meta-synthesis approach [42] to integrate findings across studies and develop higher-order interpretations.

## Results

### Search results and study selection

**Fig. 1:**
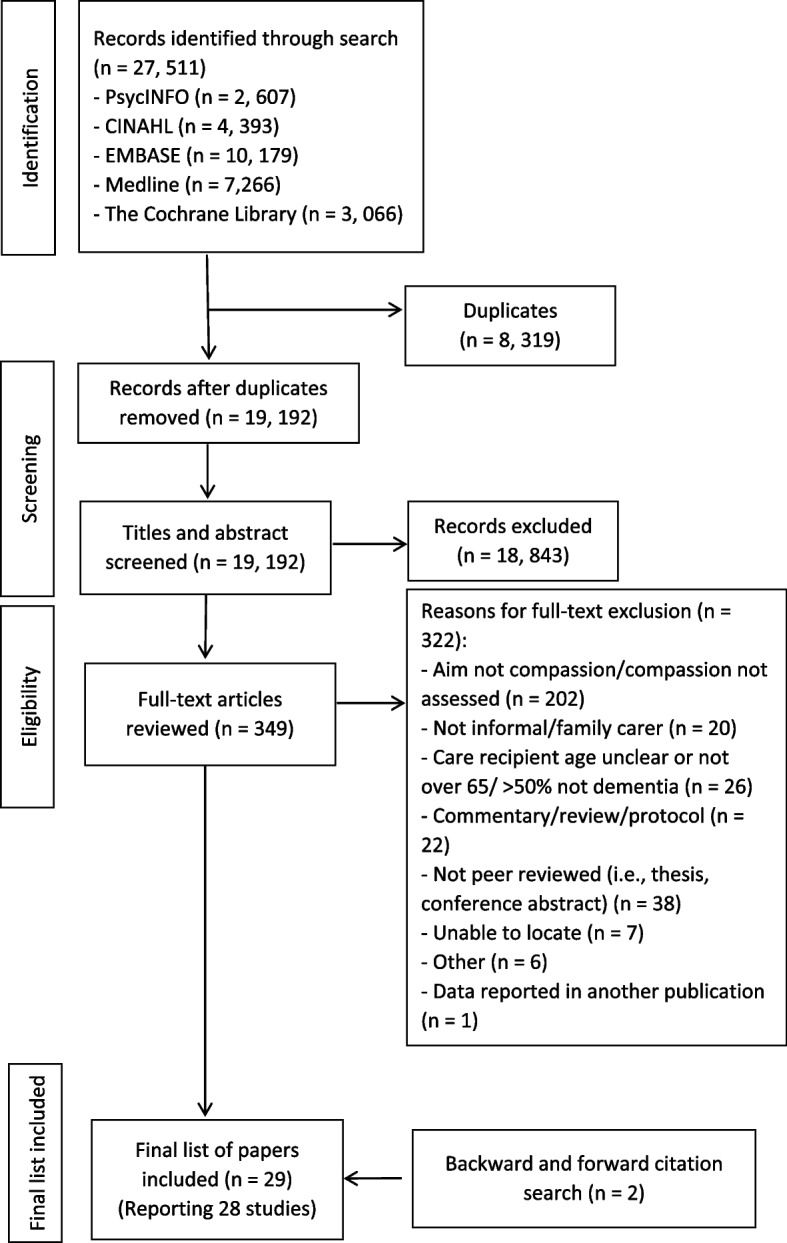
PRISMA flow diagram of study selection

A PRISMA flowchart of the screening process is presented in Fig. 1. We included 29 papers reporting 28 studies. One paper by Murfield et al., [43] was excluded because it reported data from a study cohort already represented in other included publications. Of four publications by Murfield et al., [43–46] three drew on the same study cohort: two reported quantitative findings from a large survey [43, 46], and one reported qualitative findings based on open-text responses from the same participants [45]. To avoid duplication while retaining complementary evidence, two papers from this cohort [45, 46] were included as they provided distinct types of data and one paper was excluded [43].

### Study characteristics

Tables 2 and 3 provide a summary of the characteristics of the included papers reporting quantitative (*N =* 22) and qualitative (*N =* 7) data, respectively. Studies were published between 2010 and 2025. Most were conducted in the USA (*N =* 9), Canada (*N =* 4), Australia (*N =* 3 papers reporting on two studies), Turkey (*N =* 3), UK (*N =* 3), and China (*N =* 2), and the remaining five studies in Brazil, Japan, Korea, the Netherlands and Spain.

**Table 2:** Study characteristics of studies reporting quantitative data

Study characteristics	Carer demographics	Care recipient demographics	Methods
Berk (2019) [47] Netherlands *N =* 7	Mean age: 70.8 years (SD = 8.1) Gender: 71% Female Ethnicity: NR Relationship to care recipient: 100% Partner Time caring: NR Type of carer: NR Living full-time with care recipient: NR	Mean age: 71.5 years (SD = 7.4) Gender: 29% Female Ethnicity: NR Diagnosis: 57% Alzheimer’s Disease, 29% Vascular Dementia, 14% Fronto-temporal Dementia	Theory/concept of compassion explored: Self-compassion Study design: Mixed methods interventional study (only baseline data included) Data collection: Self-completed online and paper questionnaires Setting: Community Measure of compassion: Self-compassion Scale Short Form (SCS-SF [48]) Measures of wellbeing: Relationship between compassion and wellbeing scores not explored
Bozkir (2025) [49] Turkey *N =* 382	Mean age: 38.6 years (SD = 11.2) Gender: 58% Female, 42% Male Ethnicity: NR Relationship to care recipient: NR Time caring: NR Type of carer: NR Living full-time with care recipient: NR	Mean age: 73.2 years (SD = 6.9) Gender: 55% Female, 45% Male Ethnicity: NR Diagnosis: 55.8% Cardiovascular disease/hypertension, 49% Diabetes, 15.7% Musculoskeletal diseases, 14.7% Chronic obstructive pulmonary disease/asthma, 2.9% Benign or malignant cancer	Theory/concept of compassion explored: Self-compassion Study design: Cross-sectional Data collection: Interviewer administered structured questionnaires Setting: Home care services Measure of compassion: Self-Compassion Scale (SCS [50]; Turkish version [51]) Measures of wellbeing: None
Contreras (2021) [52] UK *N =* 91	Mean age: 69.5 years (SD = 12.5) Gender: 67% Female Ethnicity: NR Relationship to care recipient: 69.3% Partner, 29.7% Offspring, 1.1% Sibling Time caring: NR Type of carer: NR Living full-time with care recipient: 73.6% Yes	Mean age: NR Gender: NR Ethnicity: NR Diagnosis: 44% Alzheimer’s Disease, 16.5% Mixed Dementia, 15.4% Vascular Dementia, 7.7% Frontotemporal Dementia, 5.5% Dementia with Lewy Bodies, 8.8% Unknown, 2.2% Other	Theory/concept of compassion explored: Self-compassion Study design: Cross-sectional Data collection: Interviewer administered structured questionnaires Setting: Carer support groups, mental health service, national online database Measure of compassion: SCS-SF [48] Measures of wellbeing: Dementia Knowledge Assessment Scale (DKAS [53]), Acceptance and Action Questionnaire-II (AAQ-II [54]), Support from other family members (single item assessing number of hours other family members provide care to care recipient), ICEpop CAPability measure for Older people (ICECAP-O [55])
Danucalov (2017) [56] Brazil N Control group = 21 N Intervention group = 25	Mean age: Control group = 53.4 years (SD = 8.2); Intervention group = 55.5 years (SD = 8.1) Gender: Control group = 90% Female, 10% Male; Intervention group = 88% Female, 12% Male Ethnicity: NR Relationship to care recipient: NR Time caring: Control group = 5.7 years (SD = 3.7); Intervention group = 4.2 years (SD = 3.3) Type of carer: NR Living full-time with care recipient: NR	Mean age: NR Gender: NR Ethnicity: NR Diagnosis: 100% Alzheimer’s Disease	Theory/concept of compassion explored: Self-compassion Study design: RCT (only baseline data included) Data collection: Self complete questionnaires Setting: Community Measure of compassion: SCS [50] Measures of wellbeing: Relationship between compassion and wellbeing scores not explored
Gallego-Alberto (2022) [57] Spain *N =* 236	Mean age: 62.3 years (SD = 12.7) Gender: 68.6% Female Ethnicity: NR Relationship to care recipient: 54.3% Offspring, 41.9% Partner, 3.8% Other Time caring: NR Type of carer: NR Living full-time with care recipient: NR	Mean age: 80.3 years (SD = 8.4) Gender: NR Ethnicity: NR Diagnosis: NR	Theory/concept of compassion explored: Carer suffering-compassion model Study design: Cross-sectional Data collection: Interviewer administered structured questionnaires Setting: Day care centres Measure of compassion: Caregiving Compassion Scale [58] Measures of wellbeing: Center for Epidemiological Studies-Depression (CES-D) scale [59], Caregiving Guilt Questionnaire [60], Caregiving Ambivalence Scale [61], single item measure assessing carer desire to place PwD in nursing home, carer’s reaction to behavioural and psychological symptoms of dementia (BPSD) as assessed by the Revised Memory and Behaviour Problems Checklist (RMBPC [62]) which is also divided into three types of problems (memory, disruptive behaviours and depressive behaviours)
Işık (2023) [63] Turkey *N =* 58	Mean age: 45.5 years (SD = 15.9) Gender: 81% Female Ethnicity: NR Relationship to care recipient: 81% family member (i.e., partner, offspring), 19% someone outside of the family Time caring: less than 2 years = 51.7%, more than 2 years = 48.3% Type of carer: NR Living full-time with care recipient:	Mean age: 77.3 years (SD = 9.4) Gender: 69% Female Ethnicity: NR Diagnosis: 100% Alzheimer’s Disease	Theory/concept of compassion explored: Compassion for others Study design: Cross-sectional Data collection: Interviewer administered structured questionnaires Setting: Elderly Living Centre Measure of compassion: SCS [50] (Turkish version [64]) Measures of wellbeing: Zarit Burden Interview [65] (Turkish version [66])
Jain (2022) [67] USA Control group *N =* 22 Intervention group *N =* 24	Mean age: Control group = 66.1 years (SD = 9.8); Intervention group = 61 years (SD = 8.4) Gender: Control group = 72.7% Female; Intervention group = 87.5% Female Ethnicity: Control group = 77.3% White, 13.6% Asian, 9.1% More than one race; Intervention group = 70.8% White, 16.7% Asian, 12.5% Black Relationship to care recipient: Control group = 54.5% Partner, 27.3% Offspring, 18.2% Other; Intervention group = 41.7% Partner, 50% Offspring, 8.3% Other Time caring: Control group = 4.2 years (SD = 2.5); Intervention group = 6.2 years (SD = 4.2) Type of carer: NR Living full-time with care recipient: Control group = 63.6% Yes; Intervention group = 83.3% Yes	Mean age: NR Gender: NR Ethnicity: NR Diagnosis: 100% Dementia	Theory/concept of compassion explored: Self-compassion Study design: RCT (only baseline data included) Data collection: Combination of self-report questionnaires and Interviewer administered structured questionnaires Setting: Community Measure of compassion: SCS-SF [48] Measures of wellbeing: Relationship between compassion and wellbeing scores not explored
James (2021) [68] USA Control group *N =* 5 Intervention group *N =* 5	Mean age: 60.3 years (SD = 7.4) Gender: 73.7% Female, 26.3% Male Ethnicity: 89.5% White/non-Hispanic, 10.5% Hispanic Relationship to care recipient: 63.2% Partner, 31.6% Offspring, 5.3% Other Time caring: 40.8 months (SD = 26.3), Range = 8–96 months Type of carer: NR Living full-time with care recipient: NR	Mean age: NR Gender: NR Ethnicity: NR Diagnosis: 100% Dementia	Theory/concept of compassion explored: Self-compassion Study design: RCT (only baseline data included) Data collection: Self complete online questionnaires Setting: Community Measure of compassion: SCS-SF [48] Measures of wellbeing: Relationship between compassion and wellbeing scores not explored
Lloyd (2019) [69] UK *N =* 73	Mean age: 67.2 years (SD = 11.5) Gender: 74% Female, 24% Male Ethnicity: 94.5% White British, 2.4% White non-European, 1.4% Other, 1.4% Did not want to say Relationship to care recipient: 69.9% Partner, 20.5% Offspring, 4.1% Sibling, 5.5% Other Time caring: NR Type of carer: NR Living full-time with care recipient: NR	Mean age: NR Gender: NR Ethnicity: NR Diagnosis: 100% Dementia	Theory/concept of compassion explored: Self-compassion Study design: Cross-sectional Data collection: Self complete postal questionnaire Setting: Post-diagnostic support service and community Measure of compassion: SCS-SF [48] Measures of wellbeing: Brief Coping Orientations to Problems Experienced scale (Brief COPE [70]; used problem- and dysfunctional-focused coping subscales), Zarit Burden Interview (ZBI) Short Form [71]
Liu (2025) [72] China Good prognosis (GP) group *N =* 91 Poor prognosis (PP) group *N =* 109	Mean age: NR Gender: NR Ethnicity: NR Relationship to care recipient: NR Time caring: NR Type of carer: NR Living full-time with care recipient: NR	Mean age: GP M 71.6 years (SD = 5.3); PP M 71.3 years (SD = 5.7) Gender: GP 46% Female, 54% Male; PP 52% Female, 48% Male Ethnicity: NR Diagnosis: 100% Chronic obstructive pulmonary disease	Theory/concept of compassion explored: Compassion fatigue and satisfaction Study design: Cross-sectional Data collection: Retrospective data collected from medical records Setting: Hospital Measure of compassion: Professional Quality of Life Scale (ProQOL) [73] Measures of wellbeing: None
Monin (2015) [74] USA *N =* 116 (58 married couple dyads)	Mean age: 71.1 years (SD = 7.8) Gender: NR Ethnicity: 94.8% White, 3.4% Black, 1.7% Asian Relationship to care recipient: 100% Partner Time caring: NR Type of carer: NR Living full-time with care recipient: NR	Mean age: 74 years (SD = 8.1) Gender: 19% Female, 81% Male Ethnicity: Same as carers Diagnosis: 100% Alzheimer’s Disease	Theory/concept of compassion explored: Compassionate love Study design: Cross-sectional Data collection: Interviewer administered structured questionnaires Setting: Community Measure of compassion: “Close other” version of compassionate love scale [75] Measures of wellbeing: ZBI short form [71], Positive appraisals of caregiving [76], CES-D (10-item version; [77]), Caregiver physical conditions [78]
Murfield (2024)^a^ [46] Australia *N =* 141	Mean age: 59.6 years (SD = 11.5) Gender: 86% Female, 14% Male Ethnicity: NR Relationship to care recipient: 54.6% Offspring, 31.2% Partner, 9.2% Relative, 2.8% Sibling, 1.4% Friend/neighbour Time caring: 6.2 years (SD = 5.5) Type of carer: 83.7% Primary, 14.9% Secondary Living full-time with care recipient: 63.1% Yes	Mean age: 81 years (SD = 9) Gender: 58.2% Female, 37.6% Male Ethnicity: NR Diagnosis: 63.1% Alzheimer’s disease or dementia, 32.6% Musculoskeletal disorders, 19.1% Cardiovascular disease, 9.2% Cancer, 7.8% Parkinson’s disease	Theory/concept of compassion explored: Compassion for self, for others and from others Study design: Cross-sectional Data collection: Self-completed online and paper questionnaires Setting: Community Measure of compassion: Compassionate Engagement and action scale (CEAS [79]) Measures of wellbeing: Mindful Attention Awareness Scale (MAAS [80]), Depression, Anxiety and Stress Scale-21 (DASS-21 [81]), Brief COPE (used problem- and emotion-focused coping) [70], Difficulties in Emotion Regulation Scale (DERS [82])
Parkman (2023) [83] USA *N =* 5	Mean age: NR (age range reported = 55–65) Gender: 100% Female Ethnicity: 100% Caucasian Relationship to care recipient: 60% Partner, 40% Offspring Time caring: 2–5 years Type of carer: NR Living full-time with care recipient: NR	Mean age: NR Gender: NR Ethnicity: NR Diagnosis: 100% Dementia	Theory/concept of compassion explored: Self-compassion Study design: Quasi-experimental, single group intervention (only baseline data included) Data collection: Self complete online questionnaires Setting: Community Measure of compassion: SCS [50] Measures of wellbeing: Relationship between compassion and wellbeing scores not explored
Pickering (2024) [84] USA *N =* 453	Mean age: 51.5 years (SD = 14) Gender: 87% Female Ethnicity: 52.2% Non-Hispanic White, 22.3% Black/African American, 23.2% Latinx/Hispanic Relationship to care recipient: 61.5% Offspring, 23% Partner Time caring: NR Type of carer: NR Living full-time with care recipient: NR	Mean age: NR Gender: NR Ethnicity: NR Diagnosis: 100% Mild cognitive impairment or dementia	Theory/concept of compassion explored: Self-compassion Study design: Cross-sectional intervention Data collection: Self complete online questionnaires Setting: Community Measure of compassion: SCS-SF [48] Measures of wellbeing: Difficulties in Emotion Regulation Scale short form (DERS-16 [85]); Daily diary of stress appraisals of Behavioural Symptoms of Dementia [86], Daily diary of elder abuse and neglect [86]
Sapra (2025) [87] USA Overall sample *N =* 133 Mindfulness group *N =* 67 Cognitive Behavioural Therapy (CBT) group *N =* 66	Mean age: Mindfulness group = 66 years (SD = 9.8); CBT group = 68.3 years (SD = 9.8) Gender: Mindfulness group = 85.1% Female, 14.9% Male; CBT group = 84.8% Female, 15.2% Male Ethnicity: Mindfulness group = 81.5% White, 16.9% African American. 1.5% Hispanic; CBT group = 81.8% White, 18.2% African American Relationship to care recipient: Mindfulness group = 70.2% Partner, 25.4% Offspring, 3% Sibling, 1.5% Other; CBT group = 70.8% Partner, 20% Offspring, 9.2% Other Time caring: NR Type of carer: NR Living full-time with care recipient: Mindfulness group = 87.9% Yes; CBT group = 92.4% Yes	Mean age: NR Gender: NR Ethnicity: NR Diagnosis: 100% Dementia	Theory/concept of compassion explored: Compassion for care recipient Study design: RCT (only baseline data included) Data collection: Interviewer administered structured questionnaires Setting: Memory and primary care clinics Measure of compassion: Compassionate love for close others [75] Measures of wellbeing: Relationship between compassion and wellbeing scores not explored
Schulz (2017)^b^ [58] USA *N =* 108	Mean age: 61 years Gender: 81% Female, 19% Male Ethnicity: 51% Hispanic, 49% African American Relationship to care recipient: NR Time caring: NR Type of carer: NR Living full-time with care recipient: NR	Mean age: NR Gender: NR Ethnicity: NR Diagnosis: 100% Dementia	Theory/concept of compassion explored: Carer suffering-compassion model Study design: Longitudinal Data collection: Interviewer administered structured questionnaires Setting: various including memory disorder clinics and community Measure of compassion: Compassion in close relationships [88, 89] Measures of wellbeing: Center for Epidemiological Studies Depression Scale (CES-S-10 [90]), care recipient physical and psychological suffering [91], intrusive thoughts using Impact of Events Scale (IES [92])
Tamura (2023) [93] Japan N Control group = 32 N Intervention group = 31	Mean age: Control group = 66.3 years (SD = 12.1); Intervention group = 63.5 years (SD = 12.7) Gender: Control group = 68.8% Female, 31.3% male; Intervention group = 74.2% Female, 25.8% Male Ethnicity: NR Relationship to care recipient: Control group = 50% Partner, 46.9% Offspring, 3.1% Other; Intervention group = 35.5% Partner, 45.2% Offspring, 9.7% daughter/son-in-law, 9.7% Other Time caring: NR Type of carer: NR Living full-time with care recipient: Control group = 78.1% Yes, 21.9% No; Intervention group = 64.5% Yes, 35.5% No	Mean age: Control group = 79.3 years (SD = 9.2); Intervention group = 81.9 years (SD = 6) Gender: Control group = 59.4% Female, 40.6% Male; Intervention group = 64.5% Female, 35.5% Male Ethnicity: NR Diagnosis: Control group = 75% Alzheimer’s Disease, 6.3% Vascular Dementia, 9.4% Dementia with Lewy Bodies, 1.6% Frontotemporal Dementia, 6.3% Other; Intervention group = 77.4% Alzheimer’s Disease, 6.5% Vascular Dementia, 6.5% Dementia with Lewy Bodies, 3.2% Frontotemporal Dementia, 6.5% Other	Theory/concept of compassion explored: Self-compassion Study design: RCT (only baseline data included) Data collection: NR Setting: Medical teaching hospital Measure of compassion: Self-compassion Reactions Inventory (SCRI-J [94]) Measures of wellbeing: Relationship between compassion and wellbeing scores not explored
Thorson-Olesen (2019) [95] USA *N =* 87 (includes 30 formal carers)	Age groups (*N =* 71): 30 to 39 (15%), 40 to 49 (27%), 50 to 59 (42%), 60 to 69 (15%) Gender: 95.4% Female, 4.6% Male Ethnicity: 95.4% White, 2.3% Black/African American, 2.3% Mixed ethnicities Relationship to care recipient: 48.3% Offspring, 34.5% Formal carer, 17.2% Partner Time caring: 6 months or longer Type of carer: NR Living full-time with care recipient: NR	Mean age: NR Gender: NR Ethnicity: NR Diagnosis: NR	Theory/concept of compassion explored: Compassion fatigue and satisfaction Study design: Cross-sectional Data collection: Self complete online questionnaires Setting: Community Measure of compassion: Professional Quality of Life Scale (ProQOL [73]) Measures of wellbeing: None
Wiita (2024)^c^ [96] UK *N =* 222	Mean age: 42.3 years (SD = 13.2), Range = 18–77 Gender: 50% Female, 49% Male, 1% Nonbinary Ethnicity: 82% White, 7.7% Asian, 3.2% African or Caribbean, 4.1% Mixed, 3.2% Other Relationship to care recipient: 62.2% Offspring, 4.1% Friend, 2.3% Partner, 1% Sibling, 30.6% Other Time caring: 5.8 years (SD = 6.8), Range 0–75 years Type of carer: NR Living full-time with care recipient: 31.1% Yes	Mean age: 78.9 years (SD = 8.3), Range = 64–90 Gender: NR Ethnicity: NR Diagnosis: 62.6% Conditions affecting mobility	Theory/concept of compassion explored: Self-compassion Study design: Experimental (only baseline data from study 3 included) Data collection: Self complete online questionnaires Setting: Community Measure of compassion: State Self-Compassion Scale long Form (SSCS-L [97]) Measures of wellbeing: Serenity, guilt and sadness subscales from the Positive and Negative Affect Schedule (PANAS [98])
Yang (2025) [99] China *N =* 264	Mean age: 53.9 years (SD = 9.9) Gender: 63.6% Female, 36.4% Male Ethnicity: NR Relationship to care recipient: 66.3% Offspring, 14.8% Son-/Daughter-in-law, 9.5% Partner, 9.4% Other Time caring: 4.3 years (SD = 3.8) Type of carer: NR Living full-time with care recipient: NR	Mean age: 81.1 years (SD = 9.9) Gender: 66.7% Female, 33.3% Male Ethnicity: NR Diagnosis: NR	Theory/concept of compassion explored: Compassion fatigue and satisfaction Study design: Cross-sectional Data collection: Interviewer administered structured questionnaires Setting: Community Measure of compassion: ProQOL (Chinese version [100]) Measures of wellbeing: Perceived Social Support Scale (Chinese version [101])
Yoo (2019) [102] Korea N Control group = 19 N Intervention group = 19	Mean age: Control group = 63.3 years (SD = 13.3); Intervention group = 65.9 years (SD = 13.4) Gender: Control group = 84.2% Female; Intervention group = 73.7% Female Ethnicity: NR Relationship to care recipient: Control group = 57.9% Partner; Intervention group = 78.9% Partner Time caring: Control group = 2 years (SD = 1.3); Intervention group = 2.9 years (SD = 1.9) Type of carer: NR Living full-time with care recipient: NR	Mean age: NR Gender: NR Ethnicity: NR Diagnosis: 100% Dementia	Theory/concept of compassion explored: Self-compassion Study design: RCT (only baseline data included) Data collection: Interviewer administered structured questionnaires Setting: Neurology and neuropsychiatry departments specialising in Dementia Measure of compassion: SCS (Korean version [103]) Measures of wellbeing: Relationship between compassion and wellbeing scores not explored
Zarei (2022) [104] Canada N Control group = 12 N Intervention group = 14	Mean age: Control group = 63 years (SD = 15); Interventio*N =* 58 years (SD = 11) Gender: Control group = 83% Female; Intervention group = 92% Female Ethnicity: NR Relationship to care recipient: Control group = 42% Partner, 58% Offspring; Interventio*N =* 21% Spouse, 79% Offspring Time caring: Control group = 4.9 years (SD = 3.6), Intervention group = 5.3 years (SD = 2.3) Type of carer: NR Living full-time with care recipient: Control group = 58.3% Yes, 41.7% No; Intervention group = 35.7% Yes, 64.3% No	Mean age: NR Gender: NR Ethnicity: NR Diagnosis: Control group = 50% Alzheimer’s Disease, 16.7% Other, 33.3% Unknown; Intervention group = 42.8% Alzheimer’s Disease, 35.7% Other, 21.5% Unknown	Theory/concept of compassion explored: Self-compassion Study design: RCT (only baseline data included) Data collection: NR Setting: Memory clinics and community Measure of compassion: SCS [50] Measures of wellbeing: Relationship between compassion and wellbeing scores not explored

*NR =* Not reported

^a^Data reported in Murfield 2021 [43] and 2024 [46] are from the same study so only data from 2024 reported which includes exploration of the relationship between compassion and other wellbeing constructs

^b^Demographic data provided by author (not reported in paper) [58]

^c^Baseline data from study 3 included (studies 1 and 2 comprise only post-intervention data) [96]

**Table 3:** Study characteristics of studies reporting qualitative data

Study characteristics	Carer demographics	Care recipient demographics	Methods
Ceylantekin (2023) [105] Turkey *N =* 10	Mean age: 58.1 (SD = 11.3) Gender: 50% Female, 50% Male Ethnicity: NR Relationship to care recipient: 70% Offspring, 30% Son/daughter-in-law Time caring: 90% 1–3 years, 10% 10 + years Type of carer: NR Living full-time with care recipient: NR	Age range: 10% 60–69 years, 40% 80–89 years, 50% 90–99 years Gender: 20% Female, 80% Male Ethnicity: NR Diagnosis: 70% Alzheimer’s Disease, 10% Chronic Obstructive Lung Disease, 10% Heart condition, 10% Paralysis	Theory/concept of compassion explored: Compassion fatigue Data collection: Semi-structured interviews Setting: Community
Day (2014) [106] USA *N =* 12	Age range: 47–65 years old Gender: 100% Female Ethnicity: 58% White, 42% Black Relationship to care recipient: 100% Offspring Time caring: mean number of years = 3.3 years, range between 6 months to 7 years Type of carer: NR Living full-time with care recipient: NR	Mean age: NR Gender: 92% Female, 8% male Ethnicity: NR Diagnosis: 100% Alzheimer’s Disease	Theory/concept of compassion explored: Compassion fatigue Data collection: Semi-structured interviews Setting: NR
Perry (2010) [10] Canada *N =* 5	Age range: 48–82 years old Gender: 80% Female, 20% male Ethnicity: NR Relationship to care recipient: 60% Offspring, 40% Partner Time caring: NR Type of carer: NR Living full-time with care recipient: NR	Age range: 80–90 years old Gender: NR Ethnicity: NR Diagnosis: NR	Theory/concept of compassion explored: Compassion fatigue Data collection: Observations and semi-structured interviews Setting: Long term care setting
Perry (2015) [107] Canada *N =* 6	Age range: NR Gender: NR Ethnicity: 83% Female, 17% male Relationship to care recipient: 83% Offspring, 17% Partner Time caring: NR Type of carer: NR Living full-time with care recipient: 100% No	Age range: NR Gender: 67% Female, 33% Male Ethnicity: NR Diagnosis: 100% Dementia	Theory/concept of compassion explored: Compassion fatigue Data collection: Observations and semi-structured interviews Setting: Long term care setting
Murfield (2022) [45] Australia *N =* 105	Mean age: 60.5 years (SD = 10.5) Gender: 89.5% Female, 10.5% Male Ethnicity: NR Relationship to care recipient: 56% Offspring, 33% Partner, 7% Relative, 3% Sibling, 1% Friend/neighbour Time caring: mean number of years = 6 years Type of carer: 86.7% Primary, 11.4% Secondary Living full-time with care recipient: 64.8% Yes, 34.3% No	Mean age: NR Gender: NR Ethnicity: NR Diagnosis: 54.3% Dementia, 29.5% Musculoskeletal Disorders, 23.8% Cardiovascular Disease, 8.6% Cancer, 5.7% Parkinson’s Disease	Theory/concept of compassion explored: Compassion for self, for others and from others Data collection: Self complete online or paper questionnaires or interviews using structured questionnaires Setting: Community
Murfield (2022) [44] Australia *N =* 14	Mean age: 62.5 years (SD = 14.4) Gender: 78.6% Female, 21.4% Male Ethnicity: NR Relationship to care recipient: 50% Partner, 50% Offspring Time caring: mean number of years = 6.5 years Type of carer: NR Living full-time with care recipient: NR	Mean age: 78.6 years (SD = 10.4) Gender: 64.3% Female, 35.7 Male Ethnicity: NR Diagnosis: 50% Alzheimer’s Disease, 28.6% Unknown, 7.1% Frontotemporal Dementia, 7.1% Dementia with Lewy Bodies, 7.1% Vascular Dementia	Theory/concept of compassion explored: Self-compassion Data collection: Semi-structured interviews and co-design groups Setting: Community
Ward-Griffin (2011) [108] Canada *N =* 20	Mean age: 52 years old Gender: 100% Female Ethnicity: NR Relationship to care recipient: 100% Offspring Time caring: NR Type of carer: NR Living full-time with care recipient: NR	Mean age: NR Gender: NR Ethnicity: NR Diagnosis: NR	Theory/concept of compassion explored: Compassion fatigue Data collection: Interviews Setting: NR

*NR* Not reported

Sample sizes ranged from 5 to 453 in the quantitative and 5 to 105 in the qualitative studies. All carers included were recruited from community settings, including carer support groups, national online databases, memory/dementia specialist clinics and long-term care settings. Where mean age of carers was reported, these ranged from 38.6 to 71.1 years. Most carers were female and either a partner or offspring to the care recipient. Where care recipient demographics were reported, mean age ranged from 71.3 to 81.9 years; most care recipients were female with a diagnosis of dementia. All data were cross-sectional (for RCTs and interventional studies, only baseline data was included) except for one study [58] which explored carer experiences of compassion in close relationships over a nine-month period.

### Methodological quality appraisal

Table 4 summarises quality appraisal scores. The majority of studies were rated as good, with five studies [10, 63, 72, 87, 105] rated as fair with scores ranging from 20 to 35.

**Table 4:** Methodological quality assessment ratings using the Hawker tool

Study	Abstract & Title	Introduction & Aims	Method & Data	Sampling	Data Analysis	Ethics & Bias	Findings/ Results	Transferability/ Generalisability	Implications & Usefulness	Overall Score (Out of 36)
Berk (2019) [47]	Fair (3)	Fair (3)	Fair (3)	Fair (3)	Good (4)	Fair (3)	Good (4)	Fair (3)	Fair (3)	Good (29)
Bozkir (2025) [49]	Good (4)	Good (4)	Good (4)	Fair (3)	Good (4)	Poor (2)	Good (4)	Fair (3)	Fair (3)	Good (31)
Ceylantekin (2023) [105]	Poor (2)	Fair (3)	Fair (3)	Fair (3)	Fair (3)	Fair (3)	Fair (3)	Fair (3)	Fair (3)	Fair (26)
Contreras (2021) [52]	Fair (3)	Fair (3)	Good (4)	Good (4)	Good (4)	Fair (3)	Good (4)	Fair (3)	Good (4)	Good (32)
Danucalov (2017) [56]	Fair (3)	Fair (3)	Fair (3)	Fair (3)	Good (4)	Fair (3)	Good (4)	Fair (3)	Fair (3)	Good (29)
Day (2014) [106]	Fair (3)	Fair (3)	Good (4)	Good (4)	Fair (3)	Very poor (1)	Good (4)	Fair (3)	Good (4)	Good (29)
Gallego-Alberto (2021) [57]	Fair (3)	Good (4)	Good (4)	Fair (3)	Good (4)	Fair (3)	Good (4)	Fair (3)	Fair (3)	Good (31)
Işık (2023) [63]	Fair (3)	Poor (2)	Fair (3)	Poor (2)	Poor (2)	Poor (2)	Poor (2)	Poor (2)	Poor (2)	Fair (20)
Jain (2022) [67]	Fair (3)	Good (4)	Fair (3)	Good (4)	Good (4)	Fair (3)	Good (4)	Good (4)	Good (4)	Good (33)
James (2021) [68]	Good (4)	Fair (3)	Good (4)	Fair (3)	Good (4)	Good (4)	Good (4)	Fair (3)	Fair (3)	Good (31)
Lloyd (2019) [69]	Good (4)	Good (4)	Good (4)	Good (4)	Good (4)	Fair (3)	Good (4)	Good (4)	Good (4)	Good (35)
Liu (2025) [72]	Fair (3)	Fair (3)	Fair (3)	Fair (3)	Fair (3)	Fair (3)	Poor (2)	Fair (3)	Poor (2)	Fair (25)
Monin (2015) [74]	Fair (3)	Fair (3)	Fair (3)	Fair (3)	Good (4)	Very poor (1)	Good (4)	Fair (3)	Good (4)	Good (28)
Murfield (2022) [45]	Fair (3)	Fair (3)	Good (4)	Good (4)	Good (4)	Good (4)	Good (4)	Good (4)	Good (4)	Good (34)
Murfield (2022) [44]	Fair (3)	Fair (3)	Good (4)	Fair (3)	Good (4)	Fair (3)	Good (4)	Fair (3)	Good (4)	Good (31)
Murfield (2024) [46]	Fair (3)	Fair (3)	Good (4)	Good (4)	Good (4)	Good (4)	Good (4)	Good (4)	Good (4)	Good (34)
Parkman (2023) [83]	Fair (3)	Good (4)	Good (4)	Fair (3)	Fair (3)	Fair (3)	Fair (3)	Fair (3)	Fair (3)	Good (29)
Perry (2010) [10]	Fair (3)	Fair (3)	Fair (3)	Poor (2)	Fair (3)	Fair (3)	Fair (3)	Poor (2)	Good (4)	Fair (26)
Perry (2015) [107]	Fair (3)	Fair (3)	Fair (3)	Fair (3)	Fair (3)	Fair (3)	Good (4)	Fair (3)	Fair (3)	Good (28)
Pickering (2024) [84]	Fair (3)	Good (4)	Good (4)	Fair (3)	Good (4)	Fair (3)	Good (4)	Fair (3)	Fair (3)	Good (31)
Sapra (2025) [87]	Good (4)	Good (4)	Fair (3)	Fair (3)	Very poor (1)	Poor (2)	Good (4)	Fair (3)	Fair (3)	Fair (27)
Schulz (2017) [58]	Good (4)	Good (4)	Good (4)	Fair (3)	Good (4)	Fair (3)	Good (4)	Fair (3)	Fair (3)	Good (31)
Tamura (2023) [93]	Good (4)	Fair (3)	Good (4)	Good (4)	Good (4)	Fair (3)	Good (4)	Good (4)	Good (4)	Good (34)
Thorson-Olesen (2019) [95]	Fair (3)	Fair (3)	Fair (3)	Poor (2)	Good (4)	Fair (3)	Good (4)	Good (4)	Good (4)	Good (30)
Ward-Griffin (2011) [108]	Good (4)	Good (4)	Fair (3)	Fair (3)	Fair (3)	Very poor (1)	Good (4)	Poor (2)	Good (4)	Good (28)
Wiita (2024) [96]	Good (4)	Good (4)	Fair (3)	Good (4)	Fair (3)	Good (4)	Good (4)	Fair (3)	Good (4)	Good (33)
Yang (2025) [99]	Fair (3)	Good (4)	Good (4)	Fair (3)	Good (4)	Good (4)	Good (4)	Fair (3)	Good (4)	Good (33)
Yoo (2019) [102]	Good (4)	Fair (3)	Good (4)	Fair (3)	Fair (3)	Fair (3)	Fair (3)	Fair (3)	Good (4)	Good (30)
Zarei (2022) [104]	Good (4)	Good (4)	Fair (3)	Fair (3)	Good (4)	Fair (3)	Good (4)	Fair (3)	Fair (3)	Good (31)

Key: Overall scoring represents Good: ≥ 28; Fair: 19–27; Poor: ≤ 18

### Integration and synthesis

#### Qualitative study findings

The majority of studies reporting qualitative data explored the impact of caring and the experience of compassion fatigue [10, 105–108] and two studies from the same authors explored carers’ experiences of compassion for self, for others and compassion from others [44, 45]. Table 3 provides a summary of the concepts explored.

These seven studies used qualitative methods to explore the experience of compassion from carers’ perspectives. Through a meta-ethnographic approach to the qualitative data, we identified four interrelated themes that explain carers’ perceptions of compassion, how it is enacted in caregiving experience, and what enables or constrains its maintenance over time.

##### Carers understand compassion as relational and other-directed

Across studies, compassion was primarily understood as an outward-facing, relational practice, grounded in love, empathy, and moral responsibility toward the care recipient. Carers rarely conceptualised compassion as something that could or should be directed toward themselves. In Murfield et al., [44], carers often struggled with the concept of compassion applied inwardly, instead situating compassion firmly in their caregiving role.

*“Self-compassion? I’m compassionate about my wife. I’m compassionate, but self-compassion? That’s the one that beat me.” * [44]

Similarly, compassion was understood as enacted through actions directed toward the care recipient, rather than through inward emotional awareness or as a way of regulating emotions.

*“I reckon in looking after Mum I am looking after myself because I want to do it. Now, to me, that’s nourishing me because I can see the benefit.” * [44]

In Ceylantekin et al., [105], carers described compassion as love, pity, and empathic concern rooted in family bonds and cultural or moral expectations:

*“I care *for* him because I love him very much.” * [105]

*“My *feeling* of compassion is too much, my eyes get filled with tears as I look at him, I cannot stand his suffering pain” * [105]

Collectively, these findings indicate that carers experience compassion as a moral and emotional commitment to another, rather than as a self-regulatory or reciprocal process.

##### Compassion is cultivated and maintained through attachment, meaning, and moral identity

Carers reported that their experience of compassion was facilitated by strong emotional attachment, prior relationship quality with the care recipient, and the meaning carers attributed to caregiving. Across studies, love and relational closeness were central to carers’ motivation and capacity to remain compassionate.

In Day et al., [106], adult daughters consistently emphasised their identity as daughters as a source of compassion and described moments of emotional closeness and fulfilment that reinforced compassion:

*We... crawled in bed together. Just because it made me feel like I was loved. And I was with my mama. And I miss not having her around like she used to be *[106].

Spiritual and moral meaning further enabled carers to maintain compassion:

*“I feel too strong and well during patient care. God gives me strength since I care for my patient, I feel tired when I come home” * [105]

*“I feel happy that I am gaining merit.” * [105]

For nurse-daughters in Ward-Griffin et al., [108], professional identity and advocacy reinforced compassionate commitment:

*“I also tended to take on that job [caring for father] because I wanted my dad to have the best care that he could get” * [108]

These findings suggest that compassion is maintained when the caring role aligns with carers’ values, identities, and sense of purpose, providing emotional meaning that offsets some of the demands of care.

##### Emotional over-identification and unrelenting responsibility undermine compassion

While attachment facilitated compassion, excessive emotional identification with the care recipient’s suffering emerged as a key barrier to maintaining compassion. Across studies, carers described absorbing the distress of the care recipient, blurring emotional boundaries.

*“When I saw my parents suffering, I suffered. I think that is the difference. I find with my clients that I am able to step back and be more of a tour guide.” * [108]

*“I think it’s because of the emotional factor, you’re subjective. You’re not objective. And we had such a close relationship as a mother and daughter, that all of a sudden, I saw her changing. She became an elderly lady and I didn’t like it…it is very difficult to look after her when you’re so emotionally attached” * [108]

Adult daughters in Day et al., [106] similarly described heightened vigilance and fear of causing distress.

*“I’m more concerned I might say or do something that might upset her.” * [106]

Alongside emotional over-identification, carers experienced continuous responsibility and hypervigilance when the care recipient is cared for by others, which further impacted their experience of compassion. In Perry and Edwards [107], carers described an inability to detach from their caring role.

*“I can’t let up. I can’t turn my back for a minute and I get disappointed in the care.” * [107]

These findings indicate that compassion becomes increasingly difficult to maintain when carers are emotionally immersed in suffering without opportunities for psychological or practical distance.

##### Fatigue, guilt, and lack of external support act as barriers to cultivating and maintaining compassion

Structural and emotional constraints consistently undermined carers’ ability to cultivate and maintain compassion. Chronic exhaustion and lack of respite were common across studies.

*“I think any caring role has to be very, very draining and doesn’t leave a lot of space for self-compassion. And there’s a lot of rhetoric around that and people constantly provide the advice of, oh do take care of yourself. But that is so bloody difficult. It really is. There’s not a lot of time, there’s not a lot of space and there’s very little energy for it.” * [44]

*“When I go home I still worry about him. I never get a break” * [10]

Many carers described deliberate emotional avoidance as necessary for survival within the caregiving role. Reflecting on emotions, including engaging in self-compassion, was feared to lead to emotional overwhelm.

Feelings of guilt and self-criticism further constrained compassion. The misconceptions surrounding compassion not only shaped carers’ understanding of the concept but also impeded the practice of self-directed compassion:

*“Often when you’re thinking about self-compassion you feel that it’s purposefully selfish.” * [44]

In Ward-Griffin et al., [108], guilt related to perceived caregiving failures was profound:

*“My biggest fear is that I would miss something…it was very exhausting, all that running around …I was beside myself. And then I think back, I wonder if I was so tired that I missed my dad’s complaint about the abdominal pain … I will take that guilt with me to the day I die.” * [108]

Conversely, compassion was more sustainable when carers were supported and able to share responsibility. In Murfield et al., [44], accepting help was framed as compassionate:

*“I get care workers and have strangers living in my house from overseas because that helps take care of things around the house that I can or can’t do. And allowing people into your life that do want to be there…to me, that’s a form of compassion.” * [44]

Support from family and friends, as well as connecting with others who have similar caregiving experiences, was perceived as protective against compassion fatigue.

*“My sisters are definitely [a support], we support each other and many, many friends have parents going through different situations and a couple of staff at work—very similar situation so we do get support and you’re feeling like you’re not alone dealing with it and lots of people are going through it.” * [108]

#### Quantitative studies measuring different concepts of compassion

Studies reporting quantitative data explored a number of different concepts of compassion using various tools (Table 5 provides a summary).

**Table 5:** Summary of the measures of compassion used in included quantitative studies

Included study	1) Concept measured 2) Measure name and reference to original development study	Subscales	N of items	Rating scale	Interpretation of scores	Population employed in original measure development
Bozkir^a^ Danucalov Parkman Yoo^b^ Zarei	1) Self-compassion 2) Self-compassion Scale (SCS) [50]	Six subscales: 1) Self-Kindness; 2) Self-Judgment; 3) Common Humanity; 4) Isolation; 5) Mindfulness; 6) Over-identification	24/ 26	1 to 5 (Almost never to almost always)	Higher scores = high compassion	University students and participants practicing Buddhist meditation (mean ages ranged from 20 to 47)
Berk Contreras Jain James Lloyd Pickering	1) Self-compassion 2) Self-compassion Scale-Short form (SCS-SF) [48]	Six subscales: 1) Self-Kindness; 2) Self-Judgment; 3) Common Humanity; 4) Isolation; 5) Mindfulness; 6) Over-identification	12	1 to 5 (Almost always to almost never)	Higher scores = high compassion	University students and general population (mean ages of three samples ranged 18–33)
Wiita	1) Self-compassion 2) State Self-Compassion Scale long Form (SSCS-L) [97]	Six subscales: 1) Self-Kindness; 2) Self-Judgment; 3) Common Humanity; 4) Isolation; 5) Mindfulness; 6) Over-identification	18	1 to 5 (not at all true for me to very true for me)	Higher scores = high compassion	University students and general population (mean ages of three samples ranged 18–74)
Tamura	1) Self-compassion 2) The Self-Compassionate Reactions Inventory—Japanese version (SCRI-J) [94]	N/A	8	4 responses: 2 self-compassionate and 2 not self-compassionate	Options to select two of the four responses available and number of self-compassionate responses selected used as an indicator of self-compassion (higher frequency of self-compassionate reasons selected = high self-compassion)	University students aged 17–22
Murfield	1) Compassion for self, for others and from others 2) Compassionate engagement and action scales (CEAS) [79]	Three individual scales (each scale produces total score and two subscale scores representing compassionate engagement and compassionate actions): 1) Compassion for self; 2) Compassion for others; 3) Compassion from others	39	1 to 10 (Never to always)	Higher scores = high compassion	University students and general population (mean ages ranged from 20–36)
Işık^c^	1) Compassion for others 2) The Compassion Scale [109]	Four subscales: 1) Kindness; 2) Indifference; 3) Common Humanity; 4) Mindfulness	24	1 to 5 (Almost never to almost always)	Higher scores = high compassion	University students, participants practicing Buddhist meditation and general population (mean ages ranged from 20 to 47)
Monin^d^ Sapra^e^	1) Compassionate love 2) Compassionate love for close others and humanity [75]	Two scales: 1) Compassionate love for close others; 2) Compassionate love for all of humanity	21 in each scale	1 to 7 (Not at all true of me to very true of me)	Higher scores = higher compassionate love	University students (mean ages ranged from 19–20 years)
Schulz	1) Compassion in close personal relationships 2) Caring in close relationships [88, 89]	N/A	11	1 to 5 (Strongly agree to strongly disagree)	Higher scores = higher compassion	Romantic couples recruited from University (mean age = 19 years)
Gallego‐Alberto^f^	1) Feelings of compassion for care recipient 2) Caregiving Compassion scale [58]	Two subscales: 1) Distress from witnessing the care recipient suffering; 2) Motivation/ disposition for helping	10	1 to 5 (Strongly agree to strongly disagree)	Higher scores = high compassion	Romantic couples (mean age = 19)
Liu^g^ Thorson-Olesen Yang^h^	1) Compassion fatigue and satisfaction 2) Professional Quality of Life Scale (ProQoL) [73]	Two subscales: 1) Compassion satisfaction; 2) Compassion fatigue (comprises two further subscales: burnout and secondary traumatic stress)	30	1 to 5 (Never to very often)	Higher scores = higher satisfaction/fatigue	Healthcare professionals

^a^Bozkir used the Turkish version of the SCS [51]

^b^Yoo used the Korean version of the SCS [103]

^c^The Turkish version of the Compassion Scale as used by Işık comprises six subscales (and not four as per the original scale) with two additional subscales measuring Disengagement and Separation [64]

^d^Monin used the Compassionate love for close others measure

^e^Sapra did not report which version of the Compassionate love measure was used

^f^Gallego-Alberto used a version of a measure developed to assess carer compassion proposed by Schulz et al. (2017) [58] which was developed based on the Caring in close relationships measure by Feeney and colleagues [88, 89]

^g^Liu did not report which version of the ProQOL was used

^h^Yang used the Chinese version of the Professional Quality of Life Scale (ProQOL) [100]

Self-compassion was the most commonly explored concept with the majority of studies [47, 49, 52, 56, 67–69, 83, 84, 102, 104] using the Self-Compassion Scale (SCS [50]) and the Self-Compassion Scale-Short form (SCS-SF [48]). Self-compassion was also measured [93, 96] using the State Self-Compassion Scale – Long Form [97] and the Self-Compassionate Reactions Inventory – Japanese version (SCRI-J [94]). The Compassionate Engagement and Action Scale (CEAS [79]) was used in one study [46] to explore compassion for self, for others and from others.

Other concepts of compassion that were explored included compassion for others [63] using the Compassion Scale [109], compassionate love [74, 87] using the compassionate love for close others and humanity [75], compassion in close personal relationships [58] using the caring in close relationships scale [88, 89], feelings of compassion for a care recipient [57] using the Caregiving Compassion Scale [58] and compassion fatigue and satisfaction [72, 95, 99] using the Professional Quality of Life scale (ProQOL [73]).

Only one study used a tool adapted to assess compassion from a carer perspective [57], however the items were adapted from a measure of motivations for and against caring in close relationships that was developed from a population of romantic couples at university with a mean age of 19 years old [88, 89]. Therefore, no study to date has explored carer experiences of compassion using a tool developed with carers.

#### Understanding the relationship between compassion and health and wellbeing outcomes

Of the 22 studies reporting quantitative data, ten explored the relationship between constructs of compassion and health and wellbeing outcomes [46, 52, 57, 58, 63, 69, 74, 84, 96, 99], with a summary of these findings presented in Table 6. We present the findings grouped into four categories: self-compassion, compassion for others, compassion from others, and compassion satisfaction, compassion fatigue, and burnout.

**Table 6:** Compassion mean scores and quantitative data exploring relationship between compassion and health and wellbeing

Author	Compassion measure/domain m scores (SD)	Relationship between compassion and measures of wellbeing
Self-compassion as assessed using the Self-Compassion Scale		
Bozkirk [49]	SCS total score (mea*N =* 16.79, SD = 2.11, *N =* 382)	Not explored
Danucalov [56]	Intervention group SCS Total score (mea*N =* 3, SD = 0.5, *N =* 25) SCS Self-kindness (mea*N =* 3.2, SD = 1, *N =* 25) SCS Self-judgement (mea*N =* 2.6, SD = 0.9, *N =* 25) SCS Common humanity (mea*N =* 3.4, SD = 0.9, *N =* 25) SCS Isolation (mea*N =* 2.7, SD = 1, *N =* 25) SCS Mindfulness (mea*N =* 3.2, SD = 1, *N =* 25) SCS Over-identification (mea*N =* 2.8, SD = 0.9, *N =* 25) Control group SCS Total score (mea*N =* 3.1, SD = 0.5, *N =* 21) SCS Self-kindness (mea*N =* 3.4, SD = 1, *N =* 21) SCS Self-judgement (mea*N =* 3.1, SD = 0.9, *N =* 21) SCS Common humanity (mea*N =* 3.3, SD = 0.8, *N =* 21) SCS Isolation (mea*N =* 2.7, SD = 1.1, *N =* 21) SCS Mindfulness (mea*N =* 3.2, SD = 0.9, *N =* 21) SCS Over-identification (mea*N =* 3, SD = 1.1, *N =* 21)	Not explored
Parkman [83]	SCS Total score by participant *Participant 1* SCS Total score (mea*N =* 1.96, SD = 0.5, *N =* 1) *Participant 2* SCS Total score (mea*N =* 2.27, SD = 0.2, *N =* 1) *Participant 3* SCS Total score (mea*N =* 2.77, SD = 0.3, *N =* 1) *Participant 4* SCS Total score (mea*N =* 4.25, SD = 1.7, *N =* 1) *Participant 5* SCS Total score (mea*N =* 3.31, SD = 0.8, *N =* 1)	Not explored
Yoo [102]	Intervention group SCS Total score (mea*N =* 87, SD = 9.6, *N =* 19) Control group SCS Total score (mea*N =* 89.7, SD = 12.7, *N =* 19)	Not explored
Zarei [104]	Intervention group SCS Total score (mea*N =* 2.57, SD = 0.62, *N =* 14) Control group SCS Total score (mea*N =* 3.1, SD = 0.64, *N =* 12)	Not explored
Self-compassion as assessed using the Self-Compassion Scale – Short Form		
Berk [47]	SCS-SF Total score (mea*N =* 3.05, SD = 0.76, *N =* 14)	Not explored
Contreras [52]	SCS-SF Total score (mea*N =* 3.28, SD = 0.7, *N =* 91)	Correlational analyses exploring relationship between self-compassion (SCS-SF) and health and wellbeing outcomes: • Positive relationship with quality of life (ICECAP-O): *r =* 0.33, *p <* 0.05 • Negative relationship with psychological inflexibility (AAQ-II): *r =* −0.65, *p <* 0.05 • No relationship with knowledge about dementia (DKAS): *r =* 0.02, ns • No relationship with support from other family members: *r =* −0.11, ns Multiple regression model: Self-compassion (SCS-SF) does not significantly predict quality of life (ICECAP-O): β = 0.05, t(4) = 0.36, p = 0.86
Jain [67]	Intervention group SCS-SF Total score (mea*N =* 38.2, SD = 8.2, *N =* 24) Control group SCS-SF Total score (mea*N =* 34.6, SD = 7.7, *N =* 22)	Not explored
James [68]	Intervention group SCS-SF Total score (mea*N =* 31, SD = 4.53, *N =* 5) Control group SCS-SF Total score (mea*N =* 35, SD = 6.93, *N =* 5)	Not explored
Lloyd [69]	SCS-SF Total score by sex *Female participants* SCS-SF Total score (mea*N =* 37.31, SD = 8.09, *N =* 52) *Male participants* SCS-SF Total score (mea*N =* 42.89, SD = 7.72, *N =* 19) SCS-SF Total score by carer relationship to care recipient *Partner* SCS-SF Total score (mea*N =* 39.61, SD = 8.31, *N =* 51) *Offspring* SCS-SF Total score (mea*N =* 36.8, SD = 8.73, *N =* 15) *Sibling* SCS-SF Total score (mea*N =* 35, SD = 1.41, *N =* 2) *Other* SCS-SF Total score (mea*N =* 37.67, SD = 10.02, *N =* 3) SCS-SF Total score by carer age < *65 years old* SCS-SF Total score (mea*N =* 38.73, SD = 8.4, *N =* 26) *66—73 years old* SCS-SF Total score (mea*N =* 36.65, SD = 7.94, *N =* 26) *74* + *years old* SCS-SF Total score (mea*N =* 40.5, SD = 8.31, *N =* 16)	Correlational analyses exploring relationship between self-compassion (SCS-SF) and health and wellbeing outcomes: • Positive relationship with emotion-focused coping (Brief COPE): *r =* 0.30, *p <* 0.01 • Negative relationship with dysfunctional coping (Brief COPE): *r =* −0.49, *p <* 0.01 • Negative relationship with burden (ZBI): *r =* −0.54, *p <*.01 Multiple regression model: • Self-compassion (SCS-SF) significantly predicts burden (ZBI): β = −0.33, t = −5.39, *p <* 0.001 Mediational analyses: • Dysfunctional coping (Brief COPE) mediates the link between self-compassion (SCS-SF) and burden (ZBI): β = −0.23 [95%CI −0.37, −0.12], *p <* 0.05 • Emotion-focused coping (Brief COPE) does not mediate the link between self-compassion (SCS-SF) and burden (ZBI): β = 0.04 [95%CI −0.03, 0.14], ns
Pickering [84]	SCS-SF total score (mea*N =* 3.44, SD = 0.71, *N =* 453)	Multilevel-moderated mediation model • Self-compassion (SCS-SF) significantly predicts elder abuse and neglect (EAN): β = − 0.38 [97.5%CI − 0.72, 0.03], p = 0.04 • Self-compassion (SCS-SF) moderates the link between emotion dysregulation (DERS-16) and elder abuse and neglect (EAN): β = 0.12 [97.5%CI 0.07, 0.17], *p <*.001
Self-compassion as assessed using the State Self-Compassion Scale – Long Form		
Wiita [96]	SSCS-L Total score (mea*N =* 3.07, SD = 0.70, *N =* 222) SSCS-L Self-kindness (mea*N =* 2.63, SD = 0.91, *N =* 222) SSCS-L Self-judgement (mea*N =* 3.02, SD = 0.93, *N =* 222) SSCS-L Common humanity (mea*N =* 3.30, SD = 0.86, *N =* 222) SSCS-L Isolation (mea*N =* 3.09, SD = 1.04, *N =* 222) SSCS-L Mindfulness (mea*N =* 3.10, SD = 0.86, *N =* 222) SSCS-L Over-identification (mea*N =* 3.27, SD = 0.88, *N =* 222)	Correlational analyses showing the relationship between self-compassion (SSCS-L) total and sub-scale scores and serenity, guilt, and sadness (PANAS): *SSCS-L Total score* • Positive relationship with Serenity: *r =* 0.55, *p <* 0.001 • Negative relationship with Guilt: *r =* −0.63, *p <* 0.001 • Negative relationship with Sadness: *r =* −0.66, *p <* 0.001 *SSCS-L Self-kindness* • Positive relationship with Serenity: *r =* 0.49, *p <* 0.001 • Negative relationship with Guilt: *r =* −0.41, *p <* 0.001 • Negative relationship with Sadness: *r =* −0.50, *p <* 0.001 *SSCS-L Self-judgement* • Positive relationship with Serenity: *r =* 0.40, *p <* 0.001 • Negative relationship with Guilt: *r =* −0.64, *p <* 0.001 • Negative relationship with Sadness: *r =* −0.53, *p <* 0.001 *SSCS-L Common humanity* • Positive relationship with Serenity: *r =* 0.28, *p <* 0.001 • Negative relationship with Guilt: *r =* −0.33, *p <* 0.001 • Negative relationship with Sadness: *r =* −0.35, *p <* 0.001 *SSCS-L Isolation* • Positive relationship with Serenity: *r =* 0.50, *p <* 0.001 • Negative relationship with Guilt: *r =* −0.54, *p <* 0.001 • Negative relationship with Sadness: *r =* −0.71, *p <* 0.001 *• SSCS-L Mindfulness* • Positive relationship with Serenity: *r =* 0.47, *p <* 0.001 • Negative relationship with Guilt: *r =* −0.52, *p <* 0.001 Negative relationship with Sadness: *r =* −0.50, *p <* 0.001 *SSCS-L Over-identification* • Positive relationship with Serenity: *r =* 0.37, *p <* 0.001 • Negative relationship with Guilt: *r =* −0.46, *p <* 0.001 • Negative relationship with Sadness: *r =* −0.42, *p <* 0.001
Self-compassion as assessed using The Self-Compassionate Reactions Inventory		
Tamura [93]	Intervention group SCRI-J Total score (mea*N =* 12.4, SD = 3.3, *N =* 31) Control group SCRI-J Total score (mea*N =* 11.5, SD = 4.2, *N =* 32)	Not explored
Compassion for self, for others and from others as assessed using the Compassionate Engagement and Action Scales (CEAS)		
Murfied [46]	CEAS individual scales total mean scores (SD) (*N =* 141) For self subscale (mea*N =* 60.7, SD = 15.6, *N =* 141) For others subscale (mea*N =* 78.1, SD = 13.6, *N =* 141) From others subscale (mea*N =* 52.4, SD = 22.0, *N =* 141)	Correlational analyses for compassion for self (CEAS) and health and wellbeing outcomes: • Negative relationship with depression (DASS-21): *r =* −0.46, *p <*.01 • Negative relationship with anxiety (DASS-21): *r =* −0.34, *p <*.01 • Negative relationship with stress (DASS-21): *r =* −0.38, *p <*.01 • Positive relationship with mindfulness (MAAS): *r =* 0.36, *p <*.01 • Positive relationship with emotion-focused coping (Brief COPE): *r =* 0.38, *p <*.01 • Positive relationship with problem-focused coping (Brief COPE): *r =* 0.46, *p <*.01 • Negative relationship with difficulties in emotion regulation (DERS): *r =* −0.59, *p <*.01 Correlational analyses for compassion for others (CEAS) and health and wellbeing outcomes: • Positive relationship with depression (DASS-21): *r =* 0.17, *p <*.05 • Positive relationship with anxiety (DASS-21): *r =* 0.18, *p <*.05 • Positive relationship with stress (DASS-21): *r =* 0.19, *p <*.05 • Negative relationship with mindfulness (MAAS): *r =* −0.21, *p <*.05 • No relationship with emotion-focused coping (Brief COPE): *r =* 0.06, ns • No relationship with problem-focused coping (Brief COPE): *r =* 0.05, ns • No relationship with difficulties in emotion regulation (DERS): *r =* 0.08, ns Correlational analyses for compassion from others (CEAS) and health and wellbeing outcomes: • Negative relationship with depression (DASS-21): *r =* −0.39, *p <*.01 • Negative relationship with anxiety (DASS-21): *r =* −0.19, *p <*.01 • Negative relationship with stress (DASS-21): *r =* −0.22, *p <*.01 • No relationship with mindfulness (MAAS): *r =* 0.06, ns • Positive relationship with emotion-focused coping (Brief COPE): *r =* 0.36, *p <*.01 • Positive relationship with problem-focused coping (Brief COPE): *r =* 0.46, *p <*.01 • Negative relationship with difficulties in emotion regulation (DERS): *r =* −0.30, *p <*.01
Compassion for others as assessed using The Compassion Scale		
Işık [63]	Overall group (*N =* 58) CS total and subscale mean scores (SD) Total score (mea*N =* 94.6, SD = 15.83, *N =* 58) Kindness subscale (mea*N =* 16.81, SD = 3.67, *N =* 58) Indifference subscale (mea*N =* 8.5, SD = 3.52, *N =* 58) Common humanity subscale (mea*N =* 15.03, SD = 3.66, *N =* 58) Disengagement subscale (mea*N =* 8.29, SD = 3.03, *N =* 58) Mindfulness subscale (mea*N =* 16.12, SD = 3.45, *N =* 58) Separation subscale (mea*N =* 8.57, SD = 3.41, *N =* 58)	Correlational analyses: No relationship between compassion for others total and subscale scores (CS) and burden (ZBI) (p-values ranged 0.5–0.9)
Compassion for care recipient as assessed using the Compassionate Love scale		
Monin [74]	Compassionate Love Scale close other scale (*N =* 52) total score (mea*N =* 6.16, SD = 0.94, *N =* 52)	Correlational analyses: • No relationship between Carer Compassionate love and physical conditions (*r =* −0.05, ns) and depression (CES-D): *r =* −0.25, ns • Positive relationship between Carer Compassionate love and positive appraisals of caring: *r =* 0.54, *p <*.01 • Negative relationship between Carer Compassionate love and burden (ZBI): *r =* −0.47, *p <*.01 Mediational analyses: • Carer Compassionate love significantly predicts carer burden (ZBI) (β = − 0.49, *p <*.01) and carer positive appraisals of caring (β = 0.54, *p <*.01) • Carer Compassionate love close other mediates the link between care recipient Compassionate love close other and carer burden (ZBI) (β = − 1.65, [95%CI − 3.17, − 0.13], *p <*.05) • Carer Compassionate love close other mediates the link between care recipient Compassionate love close other and carer positive appraisals of caring (β = 2.55, [95%CI 0.74, 4.36], *p <*.01)
Sapra [87]	Mindfulness group Compassion for care recipient total score (mea*N =* 121.54, SD = 15.27, *N =* 67) CBT group Compassion for care recipient total score (mea*N =* 123.55, SD = 13.59, *N =* 66)	Not explored
Compassion in close personal relationships as assessed using the Caring in close relationships scale		
Schulz [58]	Baseline Total score (mea*N =* 43.38, SD = 6.57, *N =* 108) Five-month follow-up Total score (mea*N =* 42.96, SD = 6.48, *N =* 91) Nine-month follow-up Total score (mea*N =* 42.84, SD = 6.68, *N =* 73)	Moderation-mediational analyses: • Compassion in close relationships predicted carer intrusive thoughts in two mediational analyses, one exploring the association between carer perceived physical suffering and depression (β = 0.19, [95% CI 0.08, 0.30], *p <*.01) and a second exploring the association between carer perceived psychological suffering and depression (β = 0.21, [95% CI 0.08, 0.33], *p <*.01) • Compassion in close relationships and carer perceived physical suffering of care recipient interaction predicted carer intrusive thoughts (β = −0.02, [95% CI −0.04, −0.004], *p <*.05) but compassion in close relationships and carer perceived psychological suffering did not predict intrusive thoughts and thus this causal path was omitted from the final model
Feelings of compassion for care recipient as assessed using the Caregiving Compassion scale		
Gallego-Alberto [57]	Caregiving Compassion Scale (*N =* 236) total score and subscale mean scores (SD) Total score (mea*N =* 32.9, SD = 6.1, *N =* 236) Distress from witnessing care recipient suffering subscale (mea*N =* 20.3, SD = 4.5, *N =* 236) Motivation/disposition for helping subscale (mea*N =* 12.6, SD = 2.4, *N =* 236)	Correlational analyses exploring relationship between overall compassion (Caregiving Compassion Scale total score) and wellbeing constructs: • No relationship with depression (CES-D): *r =* 0.08, ns Positive relationship with guilt (Caregiving Guilt Questionnaire): *r =* 0.23, *p <*.01 • No relationship with ambivalence (Caregiving Ambivalence Scale; simultaneous experience of positive and negative emotions): *r =* 0.05, ns • No relationship with desire to place care recipient in a nursing home: *r =* −0.11, ns • Positive relationship with carer reactions to behavioural and psychological symptoms of dementia (BPSD; RMBPC): *r =* 0.16, *p <*.05 • No relationship with carer reactions to care recipient memory problems (RMBPC memory subscale): *r =* 0.08, ns • No relationship with carer reactions to care recipient disruptive behaviours (RMBPC disruptive behaviours subscale): *r =* 0.03, ns • Positive relationship with carer reactions to care recipient depressive behaviours (RMBPC depressive behaviours subscale): *r =* 0.26, *p <*.01 Correlational analyses exploring relationship between Caregiving Compassion Scale subscale assessing distress from witnessing care recipient suffering and wellbeing constructs: • No relationship with depression (CES-D): *r =* 0.11, ns Positive relationship with guilt (Caregiving Guilt Questionnaire): *r =* 0.30, *p <*.01 • No relationship with ambivalence (Caregiving Ambivalence Scale): *r =* 0.12, ns • No relationship with desire to place care recipient in a nursing home: *r =* −0.05, ns • Positive relationship with carer reactions to care recipient BPSD (RMBPC): *r =* 0.21, *p <*.01 • Positive relationship with carer reactions to care recipient memory problems (RMBPC memory subscale): *r =* 0.14, *p <*.05 • No relationship with carer reactions to care recipient disruptive behaviours (RMBPC disruptive behaviours subscale): *r =* 0.06, ns • Positive relationship with carer reactions to care recipient depressive behaviours (RMBPC depressive behaviours subscale): *r =* 0.30, *p <*.01 Correlational analyses exploring relationship between Caregiving Compassion Scale subscale assessing motivation/disposition for helping and wellbeing constructs: • No relationship with depression (CES-D): *r =* 0.00, ns • No relationship with guilt (Caregiving Guilt Questionnaire): *r =* 0.01, ns • No relationship with ambivalence (Caregiving Ambivalence Scale): *r =* −0.11, ns • Positive relationship with desire to place care recipient in a nursing home: *r =* −0.18, *p <*.01 • No relationship with carer reactions to care recipient BPSD (RMBPC): *r =* 0.01, ns • No relationship with carer reactions to care recipient memory problems (RMBPC memory subscale): *r =* −0.05, ns • No relationship with carer reactions to care recipient disruptive behaviours (RMBPC disruptive behaviours subscale): *r =* −0.02, ns • No relationship with carer reactions to care recipient depressive behaviours (RMBPC depressive behaviours subscale): *r =* 0.09, ns Hierarchical regression (factors controlled for in each model were: age, gender, time caring, daily hours caring, care recipient functional status, cognitive status and frequency of care recipient BPSD): • Distress from witnessing care recipient suffering subscale did not predict depression (CES-D) (β = 0.10, ns) • Motivation/disposition for helping subscale did not predict depression (CES-D) (β = −0.08, ns) • Distress from witnessing care recipient suffering subscale predicted guilt (Caregiving Guilt Questionnaire) (β = 0.29, *p <*.001) • Motivation/disposition for helping subscale predicted guilt (Caregiving Guilt Questionnaire) (β = −0.18, *p <*.05) • Distress from witnessing care recipient suffering subscale predicted ambivalence (Caregiving Ambivalence Scale) (β = 0.19, *p <*.05) • Motivation/disposition for helping subscale predicted ambivalence (Caregiving Ambivalence Scale) (β = −0.24, *p <*.01)
Compassion fatigue and satisfaction as assessed using the Professional Quality of Life Scale		
Liu [72]	Poor prognosis group Compassion satisfaction Total score (mea*N =* 23.87, SD = 7.66, *N =* 109) Compassion fatigue Total score (mea*N =* 23.44, SD = 6.49, *N =* 109) Burnout Total score (mea*N =* 23.59, SD = 5.57, *N =* 109) Good prognosis group Compassion satisfaction Total score (mea*N =* 21.06, SD = 7.78, *N =* 91) Compassion fatigue Total score (mea*N =* 20.98, SD = 6.15, *N =* 91) Burnout Total score (mea*N =* 21.31, SD = 5.26, *N =* 91)	None
Thorson-Olesen^a^ [95]	Partner carer (*N =* 15) Compassion satisfaction Total score (mea*N =* 33.53, SD = 5.85, *N =* 15) Compassion fatigue – Burnout Total score (mea*N =* 25.4, SD = [n/a], *N =* 15) Compassion fatigue – Secondary traumatic stress Total score (mea*N =* 26.07, SD = 7.04, *N =* 15) Offspring carer (*N =* 42) Compassion satisfaction Total score (mea*N =* 34.71, SD = 7.08, *N =* 42) Compassion fatigue – Burnout Total score (mea*N =* 28.55, SD = 7.04, *N =* 42) Compassion fatigue – Secondary traumatic stress Total score (mea*N =* 26.48, SD = 6.78, *N =* 42)	Not explored
Yang [99]	Compassion satisfaction total score (mea*N =* 31.9, SD = 7.48, *N =* 264) Compassion fatigue total score (mea*N =* 26.29, SD = 6.51, *N =* 264) Burnout total score (mea*N =* 27.23, SD = 6.04, *N =* 264)	Correlational analyses: • Positive relationship between compassion satisfaction (ProQoL) and perceived social support scale: *r =* 0.61, *p <*.01 • No relationship between compassion fatigue (ProQoL) and perceived social support scale: *r =* 0.04, ns • Negative relationship between burnout (ProQoL) and perceived social support scale: *r =* −0.41, *p <*.01 Mediation and moderation analyses: • Interaction between compassion satisfaction and social support negatively predicted burnout (β = −0.003, *p <* 0.01) and compassion fatigue (β = −0.003, *p <* 0.01) • Interaction between burnout and social support negatively predicted compassion fatigue (β = −0.003, *p <* 0.01)

*NR* Not reported, *SD* Standard deviation

^a^Only data from partner and offspring included (formal carer data not included)

##### Self-compassion

A total of five studies [46, 52, 69, 84, 96] explored the relationship between self-compassion and health and wellbeing, with most finding significant associations between higher levels of self-compassion and more favourable health and wellbeing, including: quality of life [52], mindfulness [46], serenity [96], and adaptive coping strategies (emotion-focused and problem-focused coping). Negative associations were reported with psychological inflexibility [52], burden [69], dysfunctional coping [69], emotional distress (depression, anxiety, and stress) [46], difficulties in emotion regulation [46], and negative affect (guilt and sadness) [96]. These patterns were observed across both trait and state measures of self-compassion, with moderate to strong effect sizes reported across multiple subscales.

Where associations between self-compassion and quality of life were investigated, positive correlations were observed, but self-compassion did not significantly predict quality of life in multivariable regression analyses [52]. More consistent predictive effects were identified for burden, with self-compassion significantly predicting lower burden in regression models [69]. Mediational analyses indicated that dysfunctional coping mediated the relationship between self-compassion and burden, whereas emotion-focused coping did not [69].

Finally, Contreras et al., [52] did not find associations between self-compassion and dementia knowledge or perceived support from other family members. Multilevel and moderation analyses reported a direct association between higher self-compassion and lower levels of elder abuse and neglect, and a moderating effect of self-compassion on the relationship between emotion dysregulation and elder abuse and neglect [84].

##### Compassion for others

A total of five studies examined the relationship between compassion for others and health and wellbeing [46, 57, 58, 63, 74]. Across these studies, compassion for others was conceptualised and measured in diverse ways, including compassion for others [46, 63] (assessed using the Compassionate Engagement and Action Scales [CEAS [79]] and the Compassion Scale [109]), compassionate love [74], caring in close relationships [58], and feelings of compassion towards a care recipient [57].

Across studies, findings on compassion for others varied according to how compassion was operationalised and the outcomes examined. Overall, compassion for others showed inconsistent associations with carers’ mental health, but more consistent relationships with caregiving-specific appraisals and emotional responses. Compassion for others measured using the CEAS [79] was weakly but significantly associated with higher levels of depression, anxiety and stress, and lower mindfulness, with no associations observed with coping styles or difficulties in emotion regulation [46]. In contrast, other measures of compassion for others showed no relationship with depression, including carer compassionate love [74] and the Caregiving Compassion Scale (total and subscale scores) [57], findings that were confirmed in both correlational and regression analyses.

Evidence for associations with carer burden was inconsistent. No relationship was observed between compassion for others and burden [63] when assessed using the Compassion Scale [109]. However, higher levels of carer compassionate love were consistently associated with lower burden and more positive appraisals of caregiving, and compassionate love significantly predicted these outcomes in regression and mediation models [74]. Compassionate love also mediated the relationship between care-recipient compassionate love and both carer burden and positive caregiving appraisals [74].

Two studies indicated that compassion for others characterised by emotional engagement with care-recipient suffering was associated with greater emotional costs [57, 58]. Distress from witnessing suffering was positively associated with guilt, ambivalence, and stronger emotional reactions to behavioural and psychological symptoms of dementia, particularly depressive behaviours [57]. Similarly, compassion in close relationships predicted higher levels of intrusive thoughts, and interacted with perceived physical (but not psychological) suffering to exacerbate intrusive thoughts [58].

In contrast, compassion reflecting a motivation or disposition to help showed fewer adverse associations [57]. While this concept of compassion was unrelated to depression and most caregiving outcomes, it was associated with lower guilt, lower ambivalence, and a reduced desire to place the care recipient in residential care [57].

In summary, the evidence suggests that compassion for others is not uniformly protective. While some forms of compassion (e.g., compassionate love) are associated with more positive caregiving appraisals and lower burden, compassion for others involving heightened sensitivity to suffering is more consistently linked to emotional distress. These inconsistencies highlight substantial heterogeneity in both the conceptualisation and measurement of compassion for others across studies.

##### Compassion from others

One study examined the association between compassion from others, assessed using the CEAS [79], and health and wellbeing outcomes [46]. Higher levels of compassion from others were significantly associated with lower depression, anxiety and stress, as well as fewer difficulties in emotion regulation. Compassion from others was also positively associated with both emotion-focused and problem-focused coping. No association was found between compassion from others and mindfulness [46].

##### Compassion satisfaction and fatigue and burnout

One study conceptualised the experience of compassion as compassion satisfaction, compassion fatigue and burnout [99], assessed using the Professional Quality of Life (ProQoL) Scale [73], and examined their associations with health and wellbeing outcomes. Correlational analyses showed that perceived social support was positively associated with compassion satisfaction and negatively associated with burnout but was not directly associated with compassion fatigue. Mediation and moderation analyses further indicated that social support played a protective moderating role, such that higher social support attenuated the associations between compassion satisfaction and burnout, compassion satisfaction and compassion fatigue, and between burnout and compassion fatigue.

## Discussion

### Findings

We synthesised the qualitative and quantitative evidence on family carers’ experiences of compassion when caring for older adults. We focused on barriers and facilitators to cultivating and maintaining compassion, and on associations between compassion-related constructs and carers’ health and wellbeing. Taken together, the findings highlight compassion as a complex, relational, and multidimensional phenomenon that is not uniformly protective and whose effects depend on how compassion is understood, enacted, and supported.

The use of the Hawker tool allowed for quality appraisal and comparison amongst heterogeneous study designs which was then used to inform the interpretation of these results and the confidence placed in conclusions. The majority of included studies were rated as good quality using the Hawker tool, providing a degree of confidence in the overall pattern of findings. Where studies were rated as fair, this was noted and considered when drawing conclusions—findings from these studies were interpreted more cautiously and were not used as the sole basis for any substantive conclusion. Given the exploratory aim of this review and the heterogeneity across included studies, we do not claim a definitive evidence base; rather, the synthesis reflects the current state of an emerging literature, and conclusions should be interpreted in light of the variation in methodological quality described above.

Across 22 studies reporting quantitative data, ten different measures of compassion were used. These studies conceptualised and assessed the experience of compassion in a number of ways, but broadly grouped into self-compassion, compassion for others, compassion from others and compassion satisfaction, compassion fatigue and burnout. None of the measures were developed using a population of carers with one measure (ProQoL [73]) being developed with a population of healthcare professionals with the rest being developed in populations of university students and romantic couples. Notably, therefore, there were no measures of compassion explicitly developed for or using a population of carers.

There were, however, many studies which explored the lived experience of compassion among carers for older adults. The narrative synthesis consistently showed that carers conceptualise compassion as fundamentally other-directed and relational, rooted in love, empathy, moral obligation, and identity-based commitments to the care recipient. Compassion was primarily enacted through caregiving actions rather than through emotional awareness or self-regulation, and carers rarely viewed compassion as something that could be meaningfully directed toward themselves. These findings support the view that compassion should be conceptualised within a relational framework to capture the interpersonal and relational aspect of compassion [110].

Self-compassion, where acknowledged, was often experienced as difficult, unfamiliar, or even selfish. This framing positions compassion primarily as a moral responsibility toward others, rather than as a reciprocal or self-sustaining process, and helps to explain carers’ ambivalence toward self-directed compassion observed across studies. This understanding mirrors accounts of compassion within clinical practice, where compassion has been conceptualised as a moral sentiment that motivates caring behaviours [111].

Compassion was most readily cultivated when caregiving aligned with carers’ attachment, values, and sense of purpose. Emotional closeness, spiritual meaning, and identity (e.g., as a daughter) provided a foundation for maintaining compassion despite the demands of care. These findings suggest that compassion is reinforced by meaning-making processes that allow carers to integrate caregiving into their broader life narratives. Research on carer identity types indicates that those who frame caregiving as meaningful or as a source of personal growth report better psychological wellbeing [112], highlighting that the experience of compassion may be embedded in carers’ meaning-making and alignment between values and responsibilities.

However, the same relational closeness that facilitated compassion also emerged as a key vulnerability. Emotional over-identification, and unrelenting responsibility undermined carers’ ability to maintain compassion over time. Carers described internalising the perceived suffering of the care recipient, heightened vigilance, and difficulty disengaging from the caring role even when others were providing care. These experiences mirror constructs such as empathic distress [113], indicating that compassion without emotional boundaries may increase vulnerability to psychological strain.

Structural and emotional constraints further limited carers’ capacity to cultivate and maintain compassion. Chronic fatigue, lack of respite, guilt, and limited external support were pervasive barriers. Many carers described emotional avoidance as a necessary survival strategy, fearing that attending to their own emotions or practising self-compassion would be overwhelming or indulgent. Conversely, compassion appeared more sustainable when carers were able to share responsibility, accept practical help, and access validation and support from others, reframing help-seeking as a compassionate act rather than a failure.

Quantitative findings both complemented and complicated the qualitative insights. Evidence for self-compassion was largely consistent, with higher self-compassion associated with better psychological wellbeing, more adaptive coping, lower burden, and reduced emotional distress which is in line with similar literature in healthcare professionals [114]. Although predictive effects varied across outcomes and analytical approaches, the overall pattern suggests that self-compassion functions as a psychological resource for carers, even though it is not commonly recognised or endorsed by them in qualitative accounts.

In contrast, findings for compassion for others were mixed and highly contingent on how compassion was operationalised. Compassion characterised by heightened emotional engagement with suffering was associated with greater distress, intrusive thoughts, guilt, and emotional reactivity, whereas compassion reflecting a motivational or value-based orientation to help (e.g. compassionate love) was linked to lower burden and more positive caregiving appraisals. These divergent findings mirror qualitative accounts of emotional over-identification and suggest that compassion for others is not inherently protective in caregiving contexts. Notably, this contrasts with evidence from the general population, where both self-compassion and compassion for others are consistently associated with better health and wellbeing [115, 116], underscoring the importance of understanding compassion within the specific relational, emotional, and moral contexts of caregiving.

Evidence for compassion from others, although limited to a single study, indicated consistent associations with better mental health, coping, and emotion regulation, underscoring the importance of relational and social contexts. Similarly, findings on compassion satisfaction, fatigue, and burnout highlighted the protective role of social support, suggesting that compassion-related outcomes are shaped not only by individual dispositions but also by broader interpersonal and structural conditions.

Overall, the synthesis indicates that compassion in caregiving is both a source of meaning and a potential source of vulnerability, with its impact contingent on emotional boundaries, reciprocal support, and contextual resources highlighting that the boundary between facilitator and barrier when it comes to compassion, therefore, is not always clear-cut.

### Strengths and limitations

This review has several key strengths, including a comprehensive search strategy across five different databases using backwards and forwards citation searching to identify additional studies.

We also took a broad approach to understanding compassion accounting for a range of different constructs (e.g., compassion for others, compassionate love, compassion) capturing a range of studies from the emerging literature on compassion. There was also breadth in terms of the types of research methodologies included; this review integrated both qualitative and quantitative findings, providing a nuanced picture of compassion.

However, several limitations should be noted. First, the search was restricted to peer-reviewed, English-language publications, which may have introduced both language and publication bias. Studies published in other languages or disseminated through grey literature sources — such as reports, theses, and conference proceedings — were not systematically searched and may contain relevant findings that are not captured here. Second, while the Hawker quality appraisal tool was selected for its capacity to appraise a wide range of study methodologies within a single framework—consistent with the mixed-methods scope of this review— it lacks strict instruction on how to operationalise some of the criteria and thus ultimately relies on the subjective judgment of the review author. It is possible, therefore, that bias may have been introduced via the process of quality appraisal. Third, the majority of included studies were cross-sectional, limiting causal inference regarding the relationship between compassion and wellbeing. Fourth, there was substantial heterogeneity in study populations, caregiving contexts, and outcome measures, precluding meta-analysis. Fifth, most samples were predominantly female and from high-income Western countries, limiting generalisability. Sixth, the reliance on measures not developed for carers raises concerns about whether existing tools adequately capture the lived experience of compassion in caregiving. Finally, although the review focused on carers of older adults, many studies did not report detailed information about disease stage, care intensity, or duration, which may moderate the experience of compassion.

### Implications for research, practice and policy

As an emerging field of study (with the majority of studies included in this review being published in the last decade), there are a number of future directions in which research into compassion amongst carers for older adults could be taken.

This review suggests that compassion may play a role in the psychological wellbeing of carers. Longitudinal studies would, however, be needed to further understand the nature of the relationship between compassion and wellbeing in this population. Regardless, this review has highlighted the difficulty many carers report in cultivating and maintaining compassion, particularly self-compassion. In designing interventions that support the wellbeing of carers, researchers and clinicians should consider the impact that compassion (or difficulties in cultivating compassion) has. While interventions that specifically target compassion do exist and have been shown to have a positive effect on mental wellbeing [117], those that seek to facilitate the cultivation of compassion amongst carers would need to account for the various complex ways highlighted in this review in which carers experience compassion.

This review has also highlighted the lack of a psychometrically validated measure for compassion developed explicitly with and for a population of carers. While many studies have explored compassion within this population, they have relied on and adapted measures developed in other contexts. A measure developed with carers of older adults may provide a more valid assessment of how compassion is experienced in that population. This would provide clinicians and researchers working with carers with greater insight into how compassion is experienced, cultivated and maintained over time.

## Conclusion

Compassion plays a key role in the experience of carers for older adults. A growing body of qualitative and quantitative data in this area paints a complex picture. Familial obligation and bonds facilitate the cultivation of compassion but over-identification with the suffering of care recipients could undermine compassion. While cross-sectional studies point to an association between compassion and positive health and wellbeing outcomes, carers often report difficulty experiencing compassion, particularly self-compassion. Furthermore, while self-compassion and receiving compassion from others appear protective, unbuffered compassion for others may increase vulnerability to distress. Advancing research, practice, and policy requires a shift towards an understanding of compassion that accounts for the particular way it is experienced by carers of older adults.

## Data Availability

Example search strategy and data extracted from included studies provided in manuscript. Full qualitative data can be found in the manuscripts of the included studies.

## Abbreviations

BMC: BioMed Central (journal publisher)
CEAS: Compassionate Engagement and Action Scale
CENTRAL: Cochrane Central Register of Controlled Trials
CES-D: Center for Epidemiological Studies Depression Scale
CINAHL: Cumulative Index to Nursing and Allied Health Literature
COPD: Chronic Obstructive Pulmonary Disease
COPE: Coping Orientation to Problems Experienced Inventory
DASS: Depression Anxiety Stress Scales
DERS: Difficulties in Emotion Regulation Scale
DERS-16: Difficulties in Emotion Regulation Scale - 16 Item version
EMBASE: Excerpta Medica database
NHS: National Health Service
PAACC: Mindfulness-based multicomponent caregiver intervention
PANAS: Positive and Negative Affect Schedule
PANAS-X: Positive and Negative Affect Schedule - Expanded Form
PLWD: People Living With Dementia
PRISMA: Preferred Reporting Items for Systematic Reviews and Meta-Analyses
REACH: Resources for Enhancing Alzheimer’s Caregiver Health
SCRI-J: Self-Compassionate Reactions Inventory - Japanese version
SCS: Self-Compassion Scale
SCS-SF: Self-Compassion Scale-Short Form
UK: United Kingdom
